# Spelling *Pneumocystis jirovecii*

**DOI:** 10.3201/eid1503.081060

**Published:** 2009-03

**Authors:** James R. Stringer, Charles B. Beard, Robert F. Miller

**Affiliations:** University of Cincinnati, Cincinnati, Ohio, USA (J.R. Stringer); Centers for Disease Control and Prevention, Fort Collins, Colorado, USA (C.B. Beard); University College, London, UK (R.F. Miller)

**Keywords:** nomenclature, Pneumocystis, parasitology, letter

**To the Editor:** Our 2002 article in Emerging Infectious Diseases about nomenclature changes for organisms in the genus *Pneumocystis* (*1*) has been widely cited and probably will remain a source for persons seeking information about this subject. Therefore, we need to correct an error in 1 of the species names presented in our article and in the 1999 article by Frenkel (*2*) on which our article was based. In the 1999 article, Frenkel proposed that the species of *Pneumocystis* found in humans be named to honor the Czech parasitologist, Otto Jirovec. The 1999 article was his second proposal for this change. In 1976, he first named the human pathogen *Pneumocystis jiroveci* (*3*), at which time it was classified as a protozoan and therefore named according to the International Code of Zoological Nomenclature. By 1999, it had become clear that the organisms in the genus *Pneumocystis* are fungi, which are named according to the International Code of Botanical Nomenclature (ICBN) (*4*). Differences between the International Code of Zoological Nomenclature and ICBN resulted in the realization of an error in the species epithet proposed by Frenkel in 1999, and our 2002 article contained this error. Frenkel’s 1999 article should have modified the species epithet from “jiroveci” to “jirovecii,” (ICBN Articles 32.7 and 60.11 and Rec. 60C.1b). The correct and valid name under ICBN is *Pneumocystis jirovecii*. Redhead et al. further explain the basis for this correction (*5*).
